# Glutaredoxin-1 Overexpression Enhances Neovascularization and Diminishes Ventricular Remodeling in Chronic Myocardial Infarction

**DOI:** 10.1371/journal.pone.0034790

**Published:** 2012-04-16

**Authors:** Ram Sudheer Adluri, Mahesh Thirunavukkarasu, Lijun Zhan, Nageswara Rao Dunna, Yuzo Akita, Vaithinathan Selvaraju, Hajime Otani, Juan A. Sanchez, Ye-Shih Ho, Nilanjana Maulik

**Affiliations:** 1 Molecular Cardiology and Angiogenesis Laboratory, Department of Surgery, Health Center, University of Connecticut, Farmington, Connecticut, United States of America; 2 Second Department of Internal Medicine, Kansai Medical University, Moriguchi, Japan; 3 Institute of Environmental Health Sciences, Wayne State University, Detroit, Michigan, United States of America; Virginia Commonwealth University Medical Center, United States of America

## Abstract

Oxidative stress plays a critical role in the pathophysiology of cardiac failure, including the modulation of neovascularization following myocardial infarction (MI). Redox molecules thioredoxin (Trx) and glutaredoxin (Grx) superfamilies actively maintain intracellular thiol-redox homeostasis by scavenging reactive oxygen species. Among these two superfamilies, the pro-angiogenic function of Trx-1 has been reported in chronic MI model whereas similar role of Grx-1 remains uncertain. The present study attempts to establish the role of Grx-1 in neovascularization and ventricular remodeling following MI. Wild-type (WT) and Grx-1 transgenic (Grx-1^Tg/+^) mice were randomized into wild-type sham (WTS), Grx-1^Tg/+^ Sham (Grx-1^Tg/+^S), WTMI, Grx-1^Tg/+^MI. MI was induced by permanent occlusion of the LAD coronary artery. Sham groups underwent identical time-matched surgical procedures without LAD ligation. Significant increase in arteriolar density was observed 7 days (d) after surgical intervention in the Grx-1^Tg/+^MI group as compared to the WTMI animals. Further, improvement in myocardial functional parameters 30 d after MI was observed including decreased LVIDs, LVIDd, increased ejection fraction and, fractional shortening was also observed in the Grx-1^Tg/+^MI group as compared to the WTMI animals. Moreover, attenuation of oxidative stress and apoptotic cardiomyocytes was observed in the Grx-1^Tg/+^MI group as compared to the WTMI animals. Increased expression of *p*-Akt, VEGF, Ang-1, Bcl-2, survivin and DNA binding activity of NF-κB were observed in the Grx-1^Tg/+^MI group when compared to WTMI animals as revealed by Western blot analysis and Gel-shift analysis, respectively. These results are the first to demonstrate that Grx-1 induces angiogenesis and diminishes ventricular remodeling apparently through neovascularization mediated by Akt, VEGF, Ang-1 and NF-κB as well as Bcl-2 and survivin-mediated anti-apoptotic pathway in the infarcted myocardium.

## Introduction

Glutaredoxin-1 (Grx-1) is a relatively abundant, small (12 kDa) cytosolic enzyme that regulates protein mixed disulfides through their reduction by GSH (S-glutathiolated proteins; R–SSG). Unlike Trx-1, Grx-1 does not catalyze the reduction of sulfenic acid or intra- and intermolecular disulfides, thereby efficiently catalyzing the reduction of R-SSG in the presence of NADPH and glutathione reductase [1], [2]. Several lines of evidence indicate that Grx-1 is cardioprotective by attenuating oxidant-induced cell death and apoptosis [3], [4], [5]. The extent of cardiomyocyte apoptosis is enhanced in the Grx-1 knockout mice after ischemia and is decreased in mice overexpressing Grx-1 [3]. The activities of several mediators of apoptosis like procaspase-3 and p65 have been reported to be modulated by reversible glutathionylation under the control of Grx-1 [6].

Overexpression of Grx in H9c2 cardiomyocytes is cytoprotective diminishing H_2_O_2_-induced apoptosis likely through redox regulation of Akt [4]. Similarly, Grx overexpression in HEK cells is protective and enhances the cell survival after glucose deprivation via Grx-1 complex formation with ASK1 [7]. Furthermore, elevated Grx-1 expression has been found in human coronary arteries, possibly by protecting the endothelial cells from oxidative stress in both normal and atherosclerotic vessels [8]. However, the pro-angiogenic role of endogenous Grx-1 has not been reported in myocardial infarction (MI). Hence, we have hypothesized that Grx-1 acts as pro-angiogenic molecule and lowered activity of this important intracellular redox regulator during ischemic stress results in the inhibition of neovascularization.

Targeted promotion of new functional and mature vessels capable of restoring blood flow to ischemic tissues is an attractive option in the treatment of ischemic vascular disease [9]. Accumulating evidence shows that cytokines such as VEGF and Ang-1 and transcription factors like hypoxia-inducible factor-1α act as potential therapeutic pro-angiogenic molecules in experimental models [10]. Our laboratory has recently shown that Trx-1 enhances angiogenesis in chronic [11] and diabetic MI models [12] further corroborating our earlier work which reveals that redox imbalance during ischemic stress inhibits angiogenesis [12], [13].

Angiogenesis is known to be regulated by the mutual interplay between the vascular endothelial growth factor (VEGF)/VEGF receptor (VEGFR) complex and angiopoietins/Tie-2 families [14]. As one of the most potent stimulants of neovascularization, VEGF functions by promoting the expression of other growth factors in endothelial cells [15]. Additionally, Ang-1 is a secreted glycoprotein which is essential for endothelial integrity and vessel maturation via Tie-2 phosphorylation [16], [17], while VEGF is required to initiate the formation of immature vessels [18]. Ang-1 overexpressing mice appear to form larger and more mature neovessels following ischemic injury [19]. Furthermore, as a result of its antioxidant functions, Grx-1 may play a crucial role in regulating the Akt/Bcl-2 survival pathway which plays a central signaling node involved in cell growth, proliferation, differentiation, apoptosis, and angiogenesis [20]. Bcl-2 protein belongs to the family of molecules with pro-apoptotic and anti-apoptotic activity [21]. Diminished Bcl-2 level has been reported in ischemic myocardium in vivo [22]. Under similar conditions Bax protein level is found to be upregulated [22], [23].

In view of the robust ventricular remodeling observed after myocardial infarction, the role of oxidative stress–mediated reduction in myocardial angiogenesis and the antioxidative and growth regulatory action of Grx-1, we have hypothesized that overexpression of Grx-1 in the myocardium would be beneficial in promoting neovascularization and preventing subsequent ventricular remodeling after myocardial infarction through upregulation of pro-angiogenic and anti-apoptotic molecules.

## Materials and Methods

### Experimental Animals

**Figure 1:**
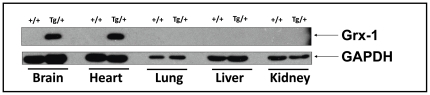
Characterization of Grx-1 transgenic mice. Expression of human Grx-1 protein in brain, heart, liver, lung and kidney of Grx-1 transgenic mice was determined by Western blot analysis. (+/+) denotes wild-type mice and (Tg/+) denotes Grx-1 transgenic mice. The anti-human Grx-1 antibodies used in the study show no cross-reactivity with the endogenous mouse Grx-1 protein.

Animal experiments were performed following the guidelines in accordance with the Guide for the Care and Use of Laboratory Animals prepared by the National Academy of Sciences and published by the National Institutes of Health (Publication No. 85-23, revised 1985). The experimental protocol was examined and approved by the Institutional Animal Care Committee (ACC # 2009-518) of UCONN Health Center (Farmington, CT, USA). Eight to 12 week-old male Grx-1^Tg/+^ overexpressing C57BL/6 background mice and respective wild-type mice were used for the study. Glutaredoxin 1 (Grx-1) transgenic mice carrying a human Grx-1 transgene driven by the human β-actin promoter were generated at Wayne State University (data not shown). The genotype of each animal was confirmed by polymerase chain reaction analysis on purified ear DNA. Expression of the human Grx-1 transgene in different organs of transgenic mice was shown by Western blot analysis (Fig. 1).

### Experimental design

Eight to 12-week-old male Grx-1^Tg/+^ and respective wild-type mice were randomized into four groups: (1) wild-type sham (WTS); (2) Grx-1^Tg/+^ Sham (Grx-1^Tg/+^S); (3) wild-type MI (WTMI) and 4) Grx-1^Tg/+^ MI (Grx-1^Tg/+^MI). MI was induced by permanent left anterior descending (LAD) coronary artery ligation. Sham groups underwent identical time-matched surgical procedure without coronary ligation. Cardiomyocyte apoptosis and oxidative stress were measured 24 h after surgical intervention. The protein expression profile for VEGF, Ang-1, Bcl-2 and survivin was determined in the left ventricular tissue (risk area/border zone of infarct) 4 days after MI. The extent of phosphorylation of Akt (p-AKT) and NF-κB DNA binding activity were measured 8 h after surgical intervention. Arteriolar density was determined 7 d after surgery. Ventricular remodeling/cardiac function was assessed by echocardiography 30 d after surgery.

### Surgical procedures

Mice were anesthetized with ketamine (100 mg/kg, ip) and xylazine (10 mg/kg, ip) dissolved in physiological saline, then orally intubated with a 22G IV catheter, and ventilated with a rodent respirator (Harvard Apparatus, Hilliston, USA). Hearts were then exposed through a left lateral thoracotomy. MI was initiated by permanent LAD ligation with 8-0 polypropylene suture viewed under a stereo zoom dissection microscope. The lungs were inflated by positive end-expiratory pressure and the chest was closed with 6.0 nylon suture. After surgery, the analgesic buprenorphine (0.1 mg/kg, sc) was given for analgesia and the animals were weaned from the respirator and then placed on a heating pad for recovery [11].

### Arteriolar density

Arteriolar density was measured 7 d after surgery by immunohistochemistry according to our previously published procedure [11].

### Echocardiography measurements

Echocardiography was performed 30 d after surgical intervention to evaluate cardiac function according to our previously published procedure [11], [12].

### Reactive Oxygen Species (ROS) detection in the heart

Mice were sacrificed 24 h after MI and horizontal heart tissue sections collected between the point of ligation and the apex were harvested and embedded in optimum cutting temperature (OCT) medium. Superoxide production in hearts 24 h after MI was detected by dihydroethidium (DHE) staining (*Invitrogen*, Carlsbad, CA, USA). Frozen heart sections (10 µM) were incubated with 10 µM DHE for 45 min at 37°C in a humidified chamber protected from light. Fluorescent images were captured using Zeiss LSM510*Meta* confocal laser scanning microscopy. The DHE fluorescence intensity was observed from 5 images/heart and 3 to 4 hearts/group. All images were processed equally and subjected to background corrections [24].

### Cardiomyocyte apoptosis

Immunohistochemical detection of apoptotic cells was carried out using TUNEL reaction using an In Situ Cell Death Detection Kit and fluorescein as per the kit protocol (Roche Diagnostics, Mannheim, Germany). The cardiomyocytes were identified with mouse monoclonal sarcomeric actin (Sigma, St. Louis, MO) followed by staining with Alexa Fluor 555 donkey anti-mouse IgG (1∶200 dilution, Invitrogen, Carlsbad, CA). Following examination of sections, images were captured using a confocal laser Zeiss LSM 510 *Meta* microscope. The number of TUNEL-positive cardiomyocytes was counted on 100 high-power fields (HPF) [11], [12].

### Western blot analysis

To determine the expression of Grx-1, p-Akt, VEGF, Ang-1 Bcl-2, Bax and survivin, standard SDS–PAGE Western blot technique was performed as described previously [11], [12]. Cytosolic and nuclear proteins were prepared according to the kit protocol of the CelLytic NuCLEAR Extraction Kit obtained from Sigma, St. Louis, MO. Protein concentration was determined using a bicinchoninic acid protein assay kit (Pierce, Rockville, IL). The proteins were separated on 10% SDS-polyacrylamide gels for p-Akt (Serine 473) (Cell Signaling Technologies, Danvers, MA), Ang-1, Bcl-2, Bax (Santa Cruz Biotechnology, Santa Cruz, CA), VEGF (R & D Systems, Minneapolis, MN) and on 14% polyacrylamide gels for survivin (Abcam, Cambridge, MA) & Grx-1 (Santa Cruz Biotechnology, Santa Cruz, CA).

### Gel-Shift analysis for NF-κB DNA binding activity

Gel-shift was performed as per the manufacturer's Instruction (Gel Shift Assay System from Promega). In brief 7 µg of the nuclear extracts following their incubation for 20 min at room temperature with ^32^P end-labeled oligonucleotides containing the putative NF-κB (5′-AGTTGAGGGGACTTTCCCAGGC-3′) (Promega, Madison, WI, USA) binding site was used. HeLaScribe Nuclear Extract (Promega, Madison, WI, USA) was used as positive control. Reaction products were resolved on 5% non-denaturing polyacrylamide gel [13].

### Statistical analysis

Results were expressed as mean ± SEM. One way ANOVA followed by Newman-Keuls multiple comparison test or unpaired “t” test (Graph Pad Prism Software) was carried out to determine differences between the mean values of all groups. The results were considered significant at P≤0.05.

## Results

### Characterization of Grx-1 transgenic mice

Western blot analysis revealed expression of the human Grx-1 protein in brain and heart of the Grx-1 transgenic mice (Fig. 1). The level of endogenous glyceraldehyde 3-pohsphate dehydrogenase (GAPDH) protein was also determined to ensure equal loading of proteins in the lysates that are prepared from the same organs of wild-type and Grx-1 transgenic mice. It should be noted that the extent of GAPDH protein is different in various organs due to the difference in the mRNA level [25]. We therefore, compared the GAPDH protein level in the same tissue (organ) sample between wild-type and Grx-1 transgenic mice.

### Grx-1 overexpression promotes neovascularization by increasing arteriolar density after myocardial infarction

**Figure 2:**
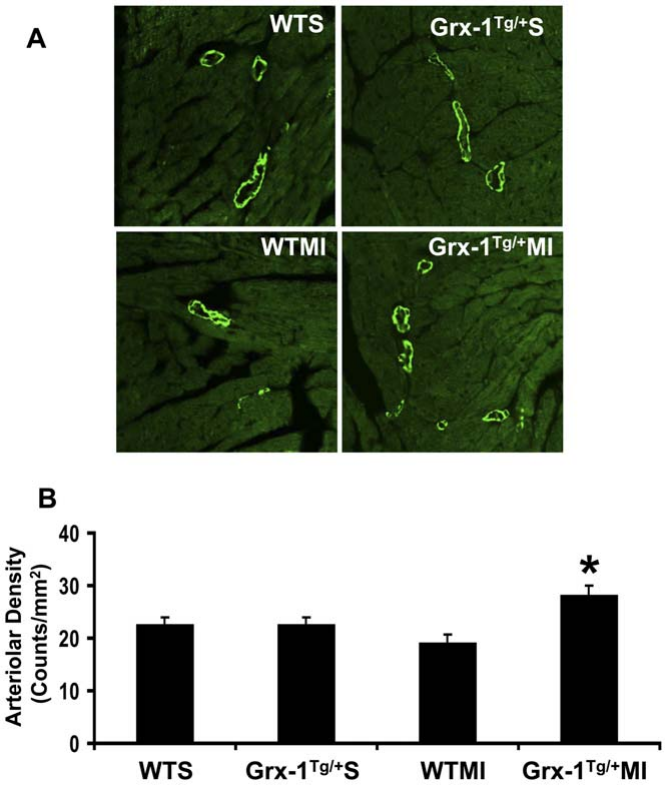
Effect of Grx-1 overexpression on arteriolar density after MI. A, Representative digital micrographs showing arteriolar density/α-smooth muscle actin immuno-staining in different experimental groups 7 days after surgical intervention. B, Quantitative analysis of arteriolar density, in counts/mm^2^. The values are mean±SEM of 3–6 animals per group. Arteriolar density was found to be significantly increased in Grx-1^Tg/+^MI compared to WTMI. WTS indicates wild-type sham; Grx-1^Tg/+^S, Grx-1 transgenic sham; WTMI, wild-type animals subjected to MI; Grx-1^Tg/+^MI, Grx-1 transgenic animals subjected to MI. *, *P*≤0.05 Grx-1^Tg/+^MI *vs.* WTMI.

Increased arteriolar density was observed in the Grx-1^Tg/+^MI group as compared to the WTMI group (28 vs 19; counts/mm^2^) (n = 3–6/group) (Fig. 2A and B). There were no significant differences in arteriolar density between the WTS and Grx-1^Tg/+^S groups. These results demonstrated that Grx-1 enhances the neovascularization by increasing the arteriolar density during ischemic stress.

### Post-ischemic ventricular remodeling is prevented by Grx-1 overexpression

**Figure 3:**
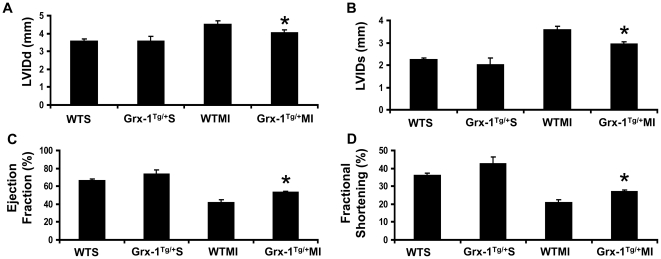
Effect of Grx-1 overexpression on cardiac function after MI. **A**, The quantitative data of left ventricular internal diameter in diastole (LVIDd); **B**, The quantitative data of left ventricular internal diameter in systole (LVIDs); **C**, ejection fraction; and **D**, fractional shortening. The data demonstrate more pronounced ventricular dysfunction in the WTMI compared to WTS and Grx-1^Tg/+^S. Grx-1 overexpression significantly improved functional parameters compared to the WTMI. Values are mean ± SEM (n = 4 per group). WTS indicates wild-type sham; Grx-1^Tg/+^S, Grx-1 transgenic sham; WTMI, wild-type animals subjected to MI; Grx-1^Tg/+^MI, Grx-1 transgenic animals subjected to MI. *, *P*≤0.05 Grx-1^Tg/+^MI *vs.* WTMI.

Left ventricular functional parameters were studied by echocardiography 30 days after coronary ligation. Left ventricular function was preserved in WTS as assessed by ejection fraction (WTS *vs.* WTMI: 67 vs. 42%) (Fig. 3C) and fractional shortening (WTS *vs.* WTMI: 36 vs 20%) (Fig. 3D) in comparison to the WTMI group. In mice overexpressing Grx-1, functional parameters significantly improved following MI when compared to the WTMI group (ejection fraction- Grx-1^Tg/+^MI vs. WTMI: 53 vs 42%; fractional shortening- Grx-1^Tg/+^MI vs. WTMI: 27 vs 20%). Furthermore, the WTMI group exhibited a progressive increase in diastolic left ventricular internal diameter (LVIDd) (Fig. 3A) and systolic LVID (LVIDs) (Fig. 3B) as compared to both the WTS, and Grx-1^Tg/+^MI LVIDs (Grx -1^Tg/+^MI *vs.* WTMI: 2.9 vs 3.6 mm) and LVIDd (Grx-1^Tg/+^MI vs. WTMI: 4.0 vs 4.5 mm) as compared to the WTMI group. Therefore, Grx-1 overexpression was associated with a progressive and significant increase in LV function as compared to the wild-type animals subjected to MI.

### Grx-1 overexpression attenuates oxidative stress and cardiomyocyte apoptosis and decreases Bax/Bcl-2 ratio after myocardial infarction

**Figure 4:**
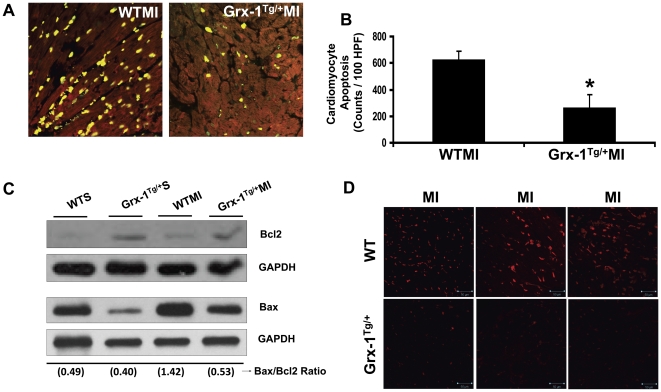
Effect of Grx-1 overexpression on the oxidative stress and cardiomyocyte apoptosis after MI. **A**, Representative digital micrographs showing cardiomyocyte apoptosis in hearts of Grx-1^Tg/+^MI and WTMI groups. **B**, Quantitative analysis of cardiomyocyte apoptosis after MI from 3–5 animals, in counts/100 high-power field (HPF). The apoptotic cardiomyocytes are significantly reduced in Grx-1^Tg/+^MI compared to WTMI. **C,** Representative Western blots showing the expression of Bcl-2, Bax, and their corresponding loading control-GAPDH in different groups. **D**, Oxidative stress (by dihydroethidium staining for O_2_^−^ production); three representative images/group show dihydroethidium-stained myocardial sections 24 h after MI. Scale bar 50 µm. Grx-1 transgenic animals subjected to MI resulted in a significant reduction in oxidative stress compared to WT animal myocardium. Dihydroethidium fluorescence was observed from 5 images per heart and 3 to 4 hearts per group. WTMI indicates wild-type animals subjected to MI; Grx-1^Tg/+^MI, Grx-1 transgenic animals subjected to MI. *, *P*≤0.05 Grx-1^Tg/+^MI *vs.* WTMI.

The extent of ROS-mediated oxidative stress was determined 24 h after MI by measuring superoxide anion (O_2_^_^) formation by utilizing dihydroethidium staining (n = 3 to 4 per group; Fig. 4D). Myocardial ROS levels were significantly decreased in the Grx-1^Tg/+^MI group as compared to the WTMI group, suggesting that Grx-1 acted as an antioxidant during ischemic stress. The extent of cardiomyocyte apoptosis (Fig. 4A), detected by using TUNEL staining in conjunction with α-sarcomeric actin significantly decreased in the Grx-1^Tg/+^MI group when compared to the WTMI (261 vs 622; counts/100 HPF) (Fig. 4B). Hence, to determine whether the extent of decrease in apoptosis as observed by TUNEL staining was accompanied by a change of the ratio between Bax and Bcl-2, we performed Western blot analysis of both Bax and Bcl-2 proteins.

Western blot analysis showed a relative increase in Bcl-2 and decrease in Bax expression in both the Grx-1^Tg/+^S and Grx-1^Tg/+^MI groups as compared to the respective wild-type sham and MI groups. The Bax/Bcl-2 ratio was found to be increased in the WTMI group as compared to the WTS group, which was similar to increased extent of apoptosis as determined by the TUNNEL staining. Overexpression of Grx-1 showed decrease in Bax/Bcl-2 ratio when compared to the WTMI group, which correlated with decreased apoptosis which was also comparable to the results obtained by TUNEL staining. These results suggested that Grx-1 acted as an anti-apoptotic molecule during ischemic stress.

### Grx-1 overexpression increases phosphorylation of Akt after MI

**Figure 5:**
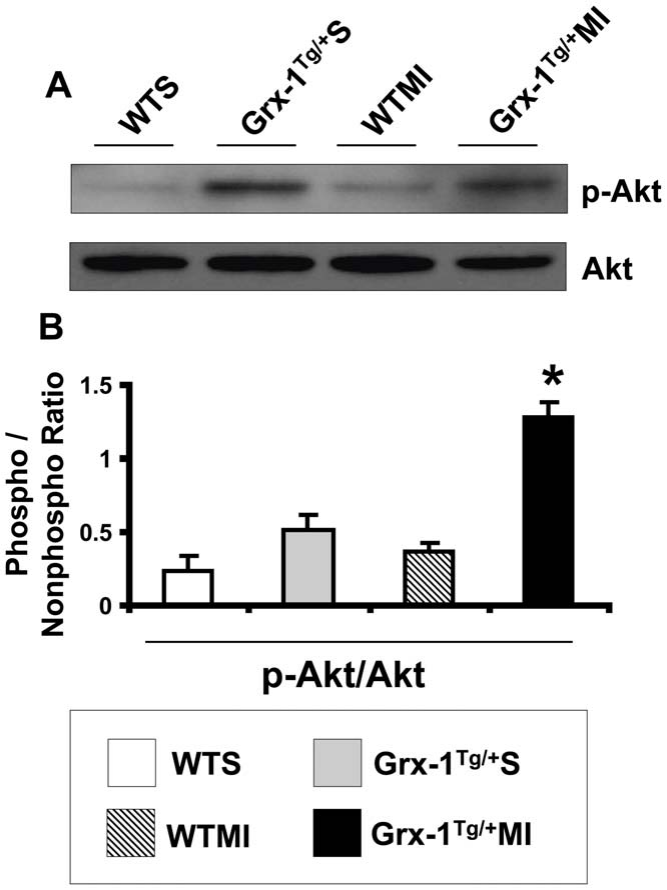
Effect of Grx-1 overexpression on the phosphorylation of Akt (p-AKT) after MI. Representative Western blots show the expression of p-Akt (A). Bar graph (B) represent the quantitative analysis and difference in the expression of p-Akt between groups, after they were normalized with corresponding non-phosphorylated protein controls, respectively in arbitrary units. The values are mean±SEM (n = 4–5 from each group). There was a significant increase in the expression of p-Akt in Grx-1^Tg/+^MI compared to the WTMI. WTS indicates wild-type sham; Grx-1^Tg/+^S, Grx-1 transgenic sham; WTMI, wild-type animals subjected to MI; Grx-1^Tg/+^MI, Grx-1 transgenic animals subjected to MI. *, *P*≤0.05 Grx-1^Tg/+^MI *vs.* WTMI.

The expression of p-Akt (Fig. 5), measured 8 h after coronary ligation significantly increased in the Grx-1^Tg/+^MI group as compared to the WTMI group, indicating the mechanism of action Grx-1 during ischemic stress probably through Akt signaling pathway.

### Grx-1 overexpression increases expression of VEGF, Ang-1, and survivin in ischemic myocardium

**Figure 6:**
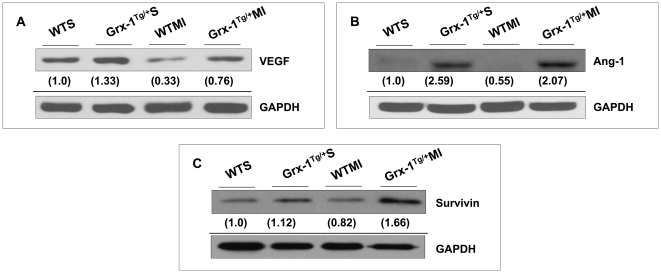
Effect of Grx-1 overexpression on VEGF, Ang-1 and survivin expression after MI. Representative Western blots showing the expression of VEGF, Ang1 and survivin, and their corresponding loading control-GAPDH in different groups. Western blot analysis revealed increased VEGF (A), Ang1, (B) and survivin (**C**) expression in Grx-1^Tg/+^MI compared to the WTMI. Numbers below the bands represent the average-fold change compared to WTS from 3–5 independent experiments. WTS indicates wild-type sham; Grx-1^Tg/+^S, Grx-1 transgenic sham; WTMI, wild-type animals subjected to MI; Grx-1^Tg/+^MI, Grx-1 transgenic animals subjected to MI.

The expression of VEGF (Fig. 6A), Ang-1 (Fig. 6B) and survivin (Fig. 6C) considerably increased in the Grx-1^Tg/+^MI group as compared to the WTMI group 4 d following infarction indicating the angiogenic and anti-apoptotic roles of Grx-1 during ischemic stress involving Akt/VEGF/Ang-1/Bcl-2/Bax and survivin.

### Grx-1 overexpression increases DNA binding activity of NF-κB during ischemic stress

**Figure 7:**
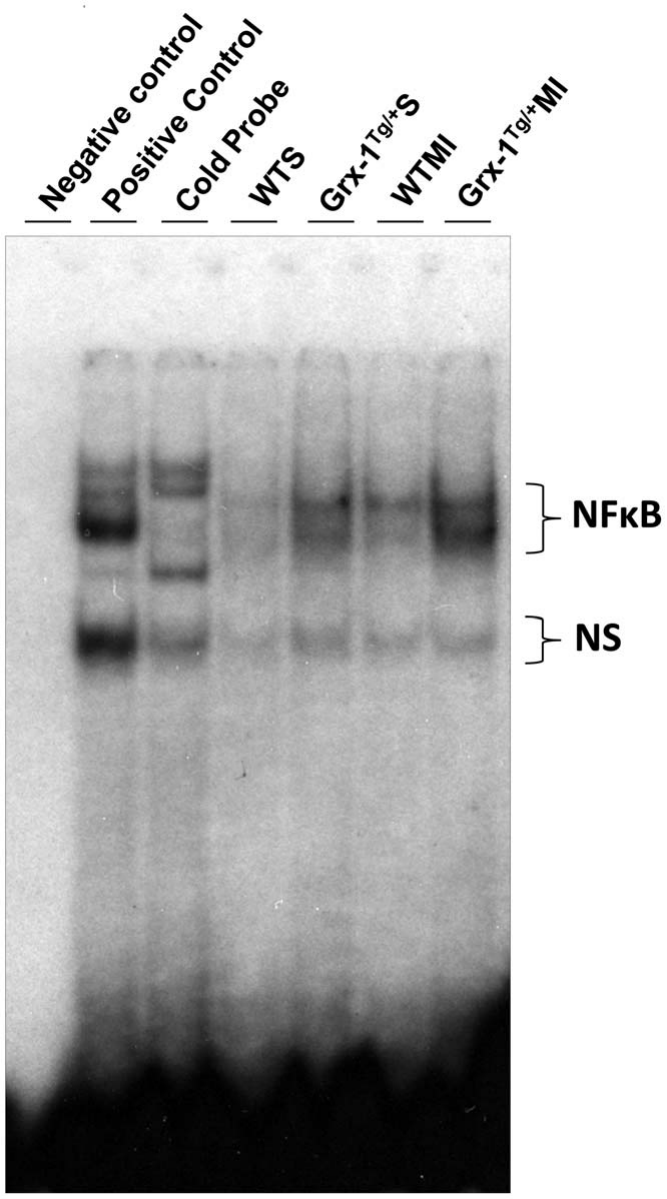
Effect of Grx-1 overexpression on DNA binding activity of NF-κB during ischemic stress. Gel-shift analysis showed increased nuclear translocation and DNA binding activity of redox transcription factor NF-κB in Grx-1^Tg/+^MI compared to WTMI (n = 3 from each group). WTS indicates wild-type sham; Grx-1^Tg/+^S, Grx-1 transgenic sham; WTMI, wild-type animals subjected to MI; Grx-1^Tg/+^MI, Grx-1 transgenic animals subjected to MI; NS, non-specific binding.

The role of Grx-1 on DNA binding activity of redox transcription factor NF-κB during ischemic stress was evaluated by EMSA/gel shift analysis. The DNA binding activity of NF-κB was significantly increased in the Grx-1^Tg/+^MI compared to the WTMI animals (Fig. 7), suggesting the possible role of Grx-1 in influencing the NF-κB expression followed by nuclear translocation and its DNA binding activity during ischemic stress.

## Discussion

Our findings suggested that, in response to MI, Grx-1: (i) promoted neovascularization through significant improvement in arteriolar density in the border zones; (ii) reduced ventricular remodeling; (iii) increased the expression of VEGF and Ang-1 (iv) increased Akt and NF-κB expression; (v) attenuated oxidative stress, reduced cardiomyocyte apoptosis and increased expression of anti-apoptotic factors Bcl-2 and survivin but decreased the expression of Bax. These current data strongly suggested that Grx-1 overexpression promotes cardiac repair after MI by increasing neovascularization and reducing ventricular remodeling through pro-angiogenic and anti-apoptotic mechanisms.

Cardiomyocyte apoptosis, subsequent ventricular remodeling, and heart failure represent important areas for therapeutic targets in cardiovascular medicine [26], [27], [28]. Strong evidence has accumulated that ROS produced during ischemic stress triggers apoptosis through a variety of mechanisms [12]. The present study demonstrated that Grx-1 overexpression significantly decreases the ROS production and cardiomyocyte apoptosis leading to improved functional and biochemical outcomes when compared to the infarcted hearts normally expressing this important intracellular redox regulator. The study also showed overexpression of Grx-1 increased both Bcl-2 and survivin and further decreased Bax/Bcl-2 ratio, which has been proposed as an important biomarker of myocardial survibility or index of apoptosis [23], [29]. We also observed that NF-κB, an important cellular regulator in normal and disease states [30] which has been implicated in the cardioprotective effects of preconditioning [31], was activated by overexpression of Grx-1. Its signaling pathways are reported to be inhibited by S-glutathionylation, thus representing other potential targets for regulation by Grx-1 [32]. Furthermore, alterations in Grx-1 activity have been linked to changes in NF-κB activity in rodent airway [33] and human kidney [34] cell lines. By demonstrating increased expression and DNA binding activity of NF-κB in the Grx-1^Tg/+^MI hearts, our current findings suggested that the decrease in apoptosis as observed in these hearts might result from both the direct antioxidant activity of Grx-1 as well as through the anti-apoptotic effects of NF-κB, Bcl-2 and survivin.

The mechanistic link between the inhibition of oxidative stress and cardioprotection by Grx-1 overexpression remains elusive. However, it is generally accepted that angiogenesis requires redox signaling [35]. It has been demonstrated that antioxidant treatment significantly reduces microvascular density in the infarcted myocardium [36], indicating that indiscriminate removal of oxygen-derived free radicals perturbs an appropriate redox condition necessary for angiogenesis and cell survival. A growing body of evidence suggests that *S*-nitrosylation plays a crucial role in angiogenesis and cardioprotection [37], [38]. Therefore, it is anticipated that overexpression of Grx-1 inhibits formation of protein mixed disulfides by eliminating oxidative stress but maintains nitroso-redox balance for *S*-nitrosylation of protein thiols leading to activation of crucial proteins involved in the angiogenesis and cardioprotection.

Endothelial tip cells sprouting at the leading edge of capillaries near the infarct zone recognize VEGF across a gradient and direct the angiogenic process towards ischemic areas [39], [40]. New vessel formation is also promoted by Ang-1/Tie-2 signaling through vessel maturation and the maintenance of endothelial integrity [17], [19], [41]. Ang-1 is also required for further maturation and remodeling of VEGF-initiated immature vessels during post-ischemic angiogenesis [14], [41] and overexpression of Ang-1 in transgenic mice has been reported to result in larger and more mature neovessel formation [19]. In our study, Grx-1 overexpression significantly increased the expression of both VEGF and Ang-1 as a result of ischemic stress resulting in an increase in arteriolar density reflecting the enhanced degree of angiogenesis.

A growing body of evidence indicates that ischemia-mediated oxidative stress modulates Akt expression and depresses the production of downstream angiogenic factors [42]. Increased Akt expression inhibits the catalytic activity of GSK-3β, thereby increasing the nuclear translocation of β-catenin and expression of its target angiogenic and anti-apoptotic genes [20]. Along with the increased VEGF and Ang-1 expression, we also observed the increased Akt-signaling in the Grx-1 transgenic animals as compared to the WT mice possibly as a result of reduced oxidative stress [42], [43] mitigated by Grx-1 overexpression.

As such, our current study suggested that Grx-1 reduced oxidative stress resulting in activation of the Akt pathway thereby up-regulating the expression of both VEGF and Ang-1 which subsequently leading to enhanced angiogenesis and neovascularization. The increased vascular density might have also resulted in increased regional perfusion, potentially accounting for the observed improvement in the left ventricular function.

In conclusion, the current findings indicated that redox imbalance inhibited normal angiogenic mechanisms which are attenuated by Grx-1 overexpression. Prompt normalization of the intracellular oxidative microenvironment following ischemic injury to the myocardium may result in lowering cell death and induction of neovascularization and vessel maturation with attendant effects on subsequent ventricular remodeling. Therapeutic approaches which enhance the expression of the antioxidant, Grx-1, may result in clinically-relevant strategies in the treatment of ischemic cardiovascular diseases.
